# Study on Vitamin K Levels in Mature Milk of Chinese Lactating Mothers

**DOI:** 10.3390/nu16193351

**Published:** 2024-10-02

**Authors:** Haiyan Wang, Zhenyu Yang, Shuxia Wang, Huwei Wu, Xuehong Pang, Yichun Hu, Xiaoguang Yang

**Affiliations:** 1 National Institute for Nutrition and Health, Chinese Center for Disease Control and Prevention, Beijing 100050, China; why0011272022@163.com (H.W.); yangzy@ninh.chinacdc.cn (Z.Y.); wangsx@ninh.chinacdc.cn (S.W.); wuhuwei@126.com (H.W.); pangxh@ninh.chinacdc.cn (X.P.); 2 Key Laboratory of Public Nutrition and Health, National Health Commission of the People’s Republic of China, Beijing 100050, China; 3 Chinese Nutrition Society, Beijing 100050, China

**Keywords:** mature milk, vitamin K, appropriate intake, infants aged 0–5 months

## Abstract

Objectives: This study sought to understand the levels of vitamin K in the mature milk of Chinese lactating mothers, thereby providing a foundation for the development of appropriate intake (AI) of vitamin K for infants aged 0–5 months. Methods: Five hundred healthy lactating mothers were selected from the mature milk bank established by the Chinese Maternal and Infant Nutrition and Health Cohort by using a simple random sample procedure. Relevant information about lactating mothers and their infants was obtained by a questionnaire survey. Vitamin K1 and vitamin K2 (MK-4 and MK-7) in mature milk were determined by liquid chromatography–tandem mass spectrometry. Results: The total concentration of vitamin K in mature milk was 4.50 (2.85–6.33) ng/mL. The concentrations of vitamin K1, vitamin K2, MK-4, and MK-7 were 2.81 (1.66–4.39) ng/mL, 1.37 (0.75–2.11) ng/mL, 1.20 (0.58–1.97) ng/mL, and 0.13 (0.08–0.19) ng/mL, respectively. The concentration of vitamin K1 was highest and the concentration of MK-7 was lowest. The concentrations of vitamin K2 and MK-4 in mature milk from the south were significantly higher than those in mature milk from the north. The total vitamin K, vitamin K2, and MK-4 concentrations in mature milk of lactating mothers residing in urban areas were higher than those in rural areas. There was a tendency for the concentration of total vitamin K and vitamin K1 to increase with the mother’s age. Moreover, the concentration of MK-4 in mature milk was highest in summer, followed by spring and winter. The levels of vitamin K1 and MK-4 in mature milk were found to be affected by lactation stage; they were highest at 91–120 days and lowest at 31–60 days. Conclusions: Based on the concentration of vitamin K in mature milk found in this study, it is recommended that the appropriate intake of VK for Chinese infants aged 0–5 months is 3.49 μg/d (2.18 μg/d for VK1 and 1.06 μg/d for VK2).

## 1. Introduction

Vitamin K (VK), an essential fat-soluble vitamin, mainly exists in the form of phylloquinone (PK or VK1) and menaquinones (VK2 or MK-n). According to the side-chain structure, VK2 is currently divided into 10 isoforms (MK-4 to MK-13) [1], among which MK-4 and MK-7 are common. VK is of critical importance for infants and young children. Infants with low VK status are at an increased risk of developing neonatal hemorrhagic disease (NHD), also known as vitamin K deficiency bleeding (VKDB) [2]. VK has a pro-coagulant function, and VK supplementation can prevent VKDB [2]. Furthermore, the potential health benefits of VK on conditions such as osteoporosis, diabetes, cardiovascular diseases, inflammation, cancer, and other diseases have attracted significant interest [3].

Breast milk is regarded as the healthiest diet for infants and the gold standard of infant nutrition recommendations as it is rich in beneficial nutrients and non-nutritional bioactive substances that can support infant survival and good development [4]. Breastfeeding is beneficial to infants, and WHO [5] recommends exclusive breastfeeding after birth, preferably for six months, and continued breastfeeding for one to two years or longer. The primary source of vitamin K for infants aged 0–5 months is breast milk. The majority of countries formulate infant formulas based on the composition of human milk, and therefore, knowledge of VK levels in mature milk is essential for establishing an appropriate intake (AI) of VK for infants in this age group. However, there is a lack of reports on the levels of VK, especially VK2, in mature milk. The objective of this study was to determine the concentrations of VK1 and VK2 in mature milk in the hope that this would provide a theoretical basis for the development of AI for VK in infants aged 0–5 months.

## 2. Materials and Methods

### 2.1. Sample Source

All the mature milk samples (breast milk produced between 15 and 180 days after delivery) were randomly selected by simple random sampling for VK concentration testing. Inclusion criteria [6] were lactating women aged 19–40 years, singleton pregnancy, non-alcoholic and non-smoking, healthy infants aged 0–5 months (free of disease), and not currently participating in any nutrition or pharmacological intervention research. Exclusion criteria [6] included mastitis, infectious disease (e.g., tuberculosis, viral hepatitis, and HIV infection), cardiovascular disease, metabolic disease (such as diabetes), mental health disorders, ‘cancer’ or other malignant or degenerative diseases, inability to answer questions, and current participation in any study related to nutrition or drug intervention.

This project was approved by the Ethics Committee of the Institute for Nutrition and Health at the Chinese Centre for Disease Control and Prevention, and all lactating mothers who participated in the project signed an informed consent form.

### 2.2. Mature Milk and Background Information Collection

One full breast was emptied using a portable automatic breast pump (HNR/X-2108Z, Shantou, Guangdong, China) in the morning (9 am to 11 am), and then stored in ice boxes or refrigerators at 4 °C. Our samples were a mixture of foremilk and hindmilk. Milk samples were gently swirled up and down 1–10 times in the bottle before being divided into five 10 mL centrifuge tubes, wrapped in aluminum foil to avoid exposure to sunlight, and delivered to the cold chain unit. They were then stored in a refrigerator at −70 °C until detection. Standardized questionnaires were used to collect data on breastfeeding mothers and their breastfed infants, including the pregnancy information and medical history of the breastfeeding mothers.

### 2.3. Materials and Reagents

MK-4 was purchased from Sigma (St. Louis, MO, USA), vitamin K1 was purchased from Aladdin (Shanghai, China), and MK-7 and vitamin K1-d4 (isotopic purity: 99.3%) were purchased from Alta (Tianjing, China). MK-4-d7 (isotopic purity: 97.5%) was purchased from Toronto Research Chemicals Inc. (Toronto, Canada), and MK-7-d7 (isotopic purity: 97.5%) was purchased from CMASS (Shanghai, China). HPLC-grade methanol, acetonitrile, and isopropanol were provided by Sigma (Shanghai, China), and HPLC-grade ethanol and formic acid (FA) were provided by Scharlab (Sentmenat, Spain). The enzymes Lipozyme TL 100 L (110 KLU/g) and Lecitase Ultra (10 KLU/g) were purchased from Novozymes A/S (Bagsaerd, Denmark). Purified water was prepared in the laboratory using the MilliQ system (Millipore, Burlington, MA, USA).

### 2.4. HPLC Conditions

VK1, MK-4, and MK-7 in milk samples were detected by the liquid chromatography–tandem mass spectrometer (LC-MS/MS). The atmospheric pressure chemical ionization source (APCI) operated in positive ion mode and the scan type was multiple reaction monitoring (MRM). Chromatographic separation was performed on a Kinetex C18 column (50 mm × 3.0 mm, 2.6 μm) (Phenomenex, Torrance, CA, USA) at a flow rate of 0.6 mL/min. The mobile phase included 0.1% FA in water (A) and 0.1% FA in methanol (B) at a gradient elution of 6.0 min.

### 2.5. Statistical Method

Lactating mothers were divided into four age groups: 19–25, 26–30, 31–35, and 36–40 years old. Residence was divided into rural and urban. Seasons were divided into spring (March–May), summer (June–August), autumn (September–November) and winter (December–February). The north and the south were bounded by the line of the Huaihe River in the Qinling Mountains, and lactation stages were divided into 15–30 d, 31–60 d, 61–90 d, 91–120 d, 121–150 d, and 151–180 d. Total VK concentration in this study was calculated as the sum of the VK1, MK-4, and MK-7 concentrations, and VK2 concentration was calculated as the sum of the MK-4 and MK-7 (the most popular reported forms of VK2) concentrations.

SAS 9.4 software (SAS Institute, Cary, NC, USA) was used for statistical analysis, and a two-tailed *p* < 0.05 was significantly different. As the data did not follow normal distribution, all indicators are expressed as P50 (P25~P75). A Mann–Whitney U test or Kruskal–Wallis rank sum test was used to test differences between groups, and Spearman correlation analysis was used to test the correlation between VK.

### 2.6. Quality Control

The quality control of vitamin K concentration determination was performed by a spiked recovery method. The recovery was 80–120%. The lower limits of quantification of VK1, MK-4, and MK-7 were 0.1 ng/mL, 0.1 ng/mL and 0.06 ng/mL, respectively.

## 3. Results

### 3.1. Basic Information

**Figure 1:**
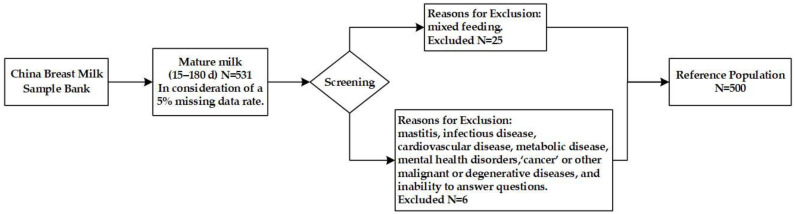
Sample screening process of the reference population.

After screening based on the exclusion criteria, 500 lactating mothers were enrolled in this study (Figure 1), 223 in urban areas and 277 in rural areas. The mean age of the lactating mothers was 26.80 years old, with the majority (81.80%) aged 19~30 years. More detailed information is presented in Table 1.

### 3.2. Concentration of VK in Mature Milk

The total VK (TVK) concentration was 4.50 (2.85–6.33) ng/mL, while the concentrations of VK1, VK2, MK-4, and MK-7 were 2.81 (1.66–4.39) ng/mL, 1.37 (0.75–2.11) ng/mL, 1.20 (0.58–1.97) ng/mL, and 0.13 (0.08–0.19) ng/mL, respectively. VK in mature milk is predominantly present in the form of VK1, followed by MK-4, with the lowest concentration observed for MK-7, and the individual variance in VK levels is considerable (Table 2).

### 3.3. Concentration of VK in Mature Milk of Different Subgroups

Significant differences were observed in the concentrations of VK1 and MK-4 in the mature milk of lactating mothers of different ages, residences, seasons, and regions.

The milk concentrations of lactating mothers aged 26–35 years were found to be significantly higher than those of mothers aged 19–25 years for both TVK and VK1. The concentrations of TVK and MK-4 were higher in urban areas than in rural areas. The concentration of VK was higher in the south than in the north. In addition, VK concentration in mature milk was significantly affected by seasonal variations, with a significantly higher concentration in spring than in fall and winter. No significant difference between groups was observed for MK-7. (Table 3).

### 3.4. Effect of Lactation Stage on VK Concentration in Mature Milk

**Figure 2:**
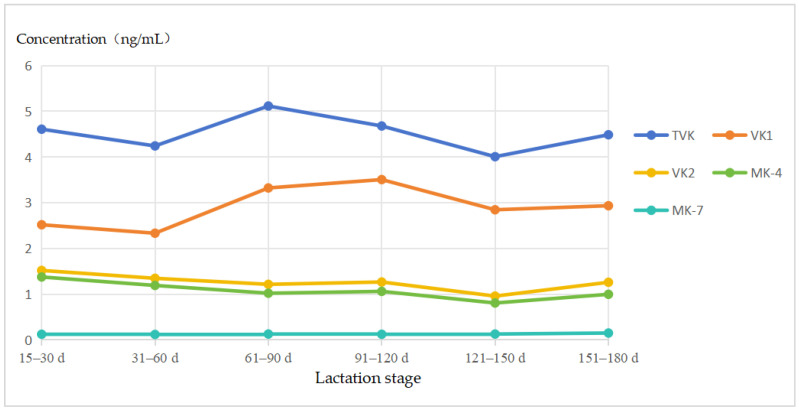
Variation trend of VK level in 15–180 d mature milk.

VK1 and MK-4 levels were significantly different in mature milk at different lactation stages. VK1 concentration in mature milk was highest at 91–120 d (3.51 ng/mL) and lowest at 31–60 d (2.33 ng/mL). VK1 concentration was significantly lower in 31–60 d mature milk than in 61–120 d and 151–180 d mature milk. The concentrations of VK2 and MK-4 in mature milk were highest at 15–30 d and lowest at 121–150 d (Figure 2). Concentrations of VK2 were significantly higher in mature milk at 15–30 d than at 31–180 d, and concentrations of MK-4 were significantly higher in mature milk at 15–30 d than at 61–180 d. There were significant differences in MK-7 concentrations in 0–2- and 3–5-month mature milks (Table 4).

### 3.5. Recommended Appropriate Intake of VK for Infants Aged 0–5 Months

The AI for VK for infants aged 0–5 months was calculated by multiplying the average breast milk intake by the VK concentration. The average breast milk intake reported in the literature is 679.60–899.00 mL/d (Table 5); combined with the data on VK concentrations obtained in our study, the corresponding AI value for VK was calculated as 3.05–4.14 μg/d (1.91–2.93 μg/d for VK1, 0.85–1.13 μg/d for VK2). We adopted the latest intake data (776.80 mL/d) based on Chinese infants. and the calculated AI value of VK was 3.49 μg/d (2.18 μg/d for VK1, 1.06 μg/d for VK2) for Chinese infants aged 0–5 months.

## 4. Discussion

VK plays a variety of health roles in lactating mothers, and its deficiency may affect the physiological health of both infants and lactating mothers. VK1 has rapidly gained use in medicine due to its clot-promoting properties [2], while supplementation of VK2 also has beneficial effects on lactating mothers and newborns, and Liu et al. found that high prenatal use of corticosteroids to prevent obstetric clinical optimization of respiratory distress syndrome (RDS), as well as cesarean section and less-than-gestational-age infants, were independently associated with VK2 deficiency [17]. VK2 supplementation improves pregnancy-associated osteoporosis and reduces the probability of vertebral compression fractures in late pregnancy and after childbirth [18]. These findings emphasize the importance of VK for pregnant women and newborns; however, there is still a lack of data on VK, especially as research on VK2 is not yet in-depth.

The average concentrations of total VK, VK1, MK-4, and MK-7 were 5.04 ± 3.28 ng/mL, 3.41 ± 2.57 ng/mL, 1.49 ± 1.39 ng/mL, and 0.15 ± 0.17 ng/mL, respectively (all above averages were recalculated for comparison with literatures). Kamao et al. [19] reported that the concentrations of VK1, MK-4, and MK-7 in mature milk of Japanese lactating mothers were 3.56 ± 2.19 ng/mL, 1.77 ± 0.68 ng/mL, and 1.19 ± 1.54 ng/mL, respectively, which are higher than those of Chinese lactating mothers. The concentrations of VK1 and MK-4 in our study were higher than those reported by Ellis et al. [20] in the mature milk of American lactating mothers (VK1, 1.3 ± 0.2 ng/mL and MK-4, 0.4 ± 0.1 ng/mL). However, in general, the concentrations of VK1 in breast milk were the highest in all of the above studies, followed by MK-4 and MK-7. And the concentration of VK, especially VK1, in mature milk has shown a wide range of individualization in all studies. This may be due to the fact that the composition of human milk is influenced by a number of factors, including the stage of lactation, genetic predisposition, maternal age, body weight and nutrition, dietary habits, and environmental factors. All of these factors result in significant inter-individual variation [6].

The results of this study indicate that the reproductive age of lactating mothers significantly affected the VK1 concentration in mature milk, and that the concentration of VK1 increases with age. However, Cao Minhui [21] and Li Sihui et al. [22] compared the main nutrients in the mature milk of lactating mothers of different reproductive ages, found that there was no statistically significant difference in the comparison of nutrient indexes in the mature milk of lactating mothers of all ages, and concluded that the age of the lactating mothers was not an influencing factor for the major nutrients in mature milk [21,22]. Nevertheless, the current body of evidence is insufficient, and further research is required to elucidate this relationship.

In addition, this study showed that there was a correlation between the concentration of MK-4 in breast milk and the place of residence of the lactating mothers. The level of MK-4 in the breast milk of lactating mothers residing in the south was found to be significantly higher than in the north, and it was higher in urban areas than in rural areas. This phenomenon may be related to dietary differences between northern and southern areas and between urban and rural areas in China. A study by Kojima et al. [23] showed that lactating mothers living in the eastern part of Japan had higher concentrations of MK-7 in their milk than those living in the western part, and hypothesized that this was related to diet, such as the frequency of natto consumption [23].

The data in the present study showed that season affects the concentration of VK in mature milk. Our study showed a difference of 15.49 ng/mL in total VK concentration in mature milk between January and August (January, 3.55 ng/mL; August, 19.04 ng/mL). The level of MK-4 in mature milk was highest in summer, and significantly higher in spring than in fall and winter. Fournier et al. [24] found that the concentration of VK in mature milk was higher in summer and the level of VK1 in mature milk was higher in August than in January, which is consistent with the results of the present study. The opposite phenomenon was found by Kojima et al., who analyzed the concentration of VK1 and VK2 in 834 mature milk samples and found that the concentrations of VK (VK1 + VK2), VK2, and MK-4 were significantly higher in mature milk in winter than in summer [23]. Seasonal variations in VK may be related to factors such as the characteristics of food intake in different seasons.

The present study found significant differences in the levels of VK1 and MK-4 in mature milk at different stages of lactation. The levels of VK1 in mature milk obtained in the present study were highest at 91–120 d (3.51 ng/mL) and lowest at 31–60 d (2.33 ng/mL). A small longitudinal study of 10 lactating mothers by Fournier et al. [24] found that the concentration of VK1 increased with the stage of lactation and that colostrum had the lowest concentration of VK1, while a higher level of VK1 was observed in mature milk. A cross-sectional study by Canfield et al. [25] determined the VK concentration of mature milk from 15 mothers and found that the VK1 level increased from 1 to 6 months postpartum, which is consistent with the results of this study. However, there are some studies that have reached opposite conclusions. Kojima et al. [23] found that total VK (VK1 + VK2), VK1, and MK-4 concentrations decreased with increasing duration of lactation from 1 to 365 d (n = 834). Meanwhile, Lambert et al. [26] also found no correlation between the concentration of VK1 in mature milk and the date of postnatal collection in the period 0–82 d postpartum (n = 26). Overall, the levels of VK in mature milk may be in dynamic change, and the relationship between VK levels and lactation stage is influenced by factors such as sample size, respondents, and different geographical regions. The studies on the effect of lactation stage on VK levels in mature milk were mainly focused on VK1, and fewer reports on VK2 were found. In our study, we found the levels of both VK2 and MK-4 were highest at 15–30 d and lowest at 121–150 d, and the levels of MK-4 were found to be significantly higher in breast milk at 15–30 d than at 61–180 d.

Currently, the AI values of VK for infants aged 0–5 months from various international organizations and nations are obtained by using the average intake of mature milk and the average concentration of VK in mature milk. The most recently established AI value for VK in infants aged 0–6 months in China is 2.0 μg/d [27], which was based on the average concentration of VK in breast milk (2.5 μg/L) and the mature milk intake of 750 mL/d reported in the literature [27].

However, most of the VK in mature milk has been reported as VK1, and the concentration of VK2 was not included, therefore the current AI value for 0–6-month-old infants established in China may be lower than their requirement. Considering the total concentration of VK in mature milk (including VK1 and VK2) in the present study (4.50 ng/mL), and the latest reported mature milk intake of 800.1 g/d (777 mL/d) [7] for infants of 0–5 months of age in China, we recommend that the AI value of VK for Chinese infants aged 0–5 months should be 3.49 μg/d (2.18 μg/d for VK1 and 1.06 μg/d for VK2), which is higher than the current recommended value of 2.0 μg/d.

The strength of this study lies in the fact that compared to previous studies, this study has a larger sample size and a broader coverage of samples, including different lactation stages of mature milk, geographical areas, which is more representative. In the meantime, we also recognize the limitations of this study. To ensure that we obtained a sufficiently large sample, we selected only mature milk from 15 to 180 days and lacked data on VK concentrations in breast milk from 0 to 15 days. In addition, other subtypes of VK2, such as MK-6, MK-9, MK-10, etc., are rarely reported in breast milk. With the limited number of breast milk samples we obtained, only the two most common subtypes, MK-4 and MK-7, were detected in this study.

## 5. Conclusions

In conclusion, the results of this study provide important data for updates on the AI recommendations for VK (or separate recommendations for VK1 and VK2) for infants aged 0–5 months in China. The VK level in mature milk was 4.50 ng/mL, and the concentrations of VK1, VK2, MK-4, and MK-7 were 2.81 ng/mL, 1.37 ng/mL, 1.20 ng/mL, and 0.13 ng/mL, respectively. We recommend that the AI value of VK for Chinese infants aged 0–5 months is 3.49 μg/d (2.18 μg/d for VK1 and 1.06 μg/d for VK2).

## Figures and Tables

**Table 1:** Basic characteristics of the study population.

	Group	N	%
Age (years)			
	19–25	206	41.20
	26–30	203	40.60
	31–35	68	13.60
	36–40	19	3.80
Residence			
	Urban	223	44.60
	Rural	277	55.40
Region			
	South	128	25.60
	North	372	74.40
BMI			
	<18.5	33	6.60
	18.5 ≤ BMI < 24	303	60.60
	24 ≤ BMI < 28	122	24.40
	BMI ≥ 28	42	8.40
Lactation stage			
	15–30 d	177	35.40
	31–60 d	88	17.60
	61–90 d	65	13.00
	91–120 d	63	12.60
	121–150 d	62	12.40
	151–180 d	45	9.00

**Table 2:** VK concentration in mature milk (ng/mL).

Index	Concentration	Range
TVK	4.50 (2.85–6.33)	0.10–24.53
VK1	2.81 (1.66–4.39)	0.02–19.88
VK2	1.37 (0.75–2.11)	0.04–15.40
MK-4	1.20 (0.58–1.97)	0.02–15.28
MK-7	0.13 (0.08–0.19)	0.01–3.17

TVK = VK1 + MK-4 + MK-7, VK2 = MK-4 + MK-7. VK concentration is expressed as P50 (P25–P75).

**Table 3:** VK concentration in different subgroups (ng/mL).

Group	N	TVK		VK1		VK2		MK-4		MK-7	
		Concentration	*p*	Concentration	*p*	Concentration	*p*	Concentration	*p*	Concentration	*p*
Total		4.50 (2.85–6.33)		2.81 (1.66–4.39)		1.37 (0.75–2.11)		1.20 (0.58–1.97)		0.13 (0.08–0.19)	
Age group (years)			0.026		0.029		0.083		0.085		0.885
19–25	206	4.00 (2.59–5.91) b		2.51 (1.52–3.66) a		1.20 (0.64–1.96)		1.04 (0.49–1.79)		0.12 (0.08–0.18)	
26–30	203	4.73 (2.85–6.68) a		3.20 (1.78–4.63) bc		1.49 (0.77–2.27)		1.28 (0.62–2.05)		0.12 (0.08–0.19)	
31–35	68	4.91 (3.39–6.74) a		3.22 (1.93–4.77) bc		1.57 (1.05–2.36)		1.37 (0.86–2.15)		0.14 (0.09–0.18)	
36–40	19	3.65 (2.36–5.90) ab		2.78 (1.26–4.19) ab		1.29 (0.61–2.60)		1.15 (0.53–2.54)		0.12 (0.10–0.19)	
Residence			0.044		0.120		<0.001		<0.001		0.494
Urban	223	4.65 (3.25–6.35)		2.95 (1.93–4.56)		1.52 (1.00–2.22)		1.35 (0.85–2.06)		0.13 (0.09–0.19)	
Rural	277	4.30 (2.38–6.31)		2.75 (1.51–4.24)		1.16 (0.56–1.99)		0.10 (0.43–1.87)		0.12 (0.08–0.19)	
Season			0.408		0.072		0.004		<0.001		0.515
Spring	104	4.49 (3.13–6.34)		2.67 (1.43–4.18)		1.63 (1.05–2.34) ac		1.50 (0.94–2.17) ac		0.11 (0.08–0.17)	
Summer	9	5.26 (2.94–13.67)		3.60 (1.57–8.34)		1.79 (1.23–2.65) ab		1.71 (1.15–2.37) ab		0.09 (0.07–0.28)	
Autumn	241	4.47 (2.82–6.56)		2.96 (1.86–4.76)		1.22 (0.62–1.95) b		1.01 (0.49–1.74) b		0.14 (0.08–0.20)	
Winter	146	4.58 (2.51–6.01)		2.69 (1.55–4.01)		1.43 (0.62–2.11) b		1.24 (0.48–1.92) b		0.13 (0.08–0.18)	
Region			0.172		0.537		0.001		<0.001		0.323
South	128	4.62 (3.06–7.01)		2.78 (1.63–4.58)		1.55 (0.98–2.35)		1.46 (0.86–2.22)		0.11 (0.08–0.17)	
North	372	4.32 (2.73–6.19)		2.84 (1.66–4.21)		1.31 (0.63–1.98)		1.13 (0.50–1.83)		0.13 (0.08–0.19)	

TVK = VK1 + MK-4 + MK-7, VK2 = MK-4 + MK-7. a, b, c statistical difference between groups. VK concentration is expressed as P50 (P25–P75).

**Table 4:** Effect of lactation stage on VK concentration in mature milk (ng/mL).

Lactation Stage	N	TVK	VK1	VK2	MK-4	MK-7
0–2 month	330	4.60 (3.02–5.27)	2.69 (1.57–4.19)	1.44 (0.88–2.27) *	1.28 (0.73–2.05)	0.12 (0.08–0.18) *
15–30 d	177	4.61 (3.14–6.38)	2.52 (1.49–4.18) bc	1.52 (1.06–2.40) a	1.38 (0.91–2.20) a	0.12 (0.08–0.19)
31–60 d	88	4.24 (2.52–5.55)	2.33 (1.54–3.76) b	1.35 (0.77–2.01) b	1.19 (0.58–1.91) ab	0.12 (0.08–0.16)
61–90 d	65	5.12 (3.07–7.34)	3.32 (1.98–5.30) ac	1.22 (0.65–2.26) b	1.02 (0.49–2.01) b	0.13 (0.08–0.19)
3–5 month	170	4.43 (2.58–6.44)	3.04 (1.69–4.91)	1.15 (0.56–1.88) *	1.00 (0.40–1.70)	0.13 (0.08–0.20) *
91–120 d	63	4.68 (2.83–6.95)	3.51 (1.78–5.52) a	1.27 (0.55–1.94) b	1.06 (0.38–1.78) b	0.12 (0.07–0.17)
121–150 d	62	4.01 (2.28–6.38)	2.85 (1.37–4.30) ab	0.96 (0.58–1.68) b	0.81 (0.49–1.60) b	0.13 (0.08–0.22)
151–180 d	45	4.49 (2.86–7.21)	2.94 (2.10–5.40) ac	1.26 (0.53–1.97) b	0.10 (0.37–1.71) b	0.15 (0.10–0.23)
*p*		0.208	0.018	0.005	0.004	0.422

TVK = VK1 + MK-4 + MK-7, VK2 = MK-4 + MK-7. a, b, c statistical difference between groups, *p* < 0.05. VK concentration is expressed as P50 (P25–P75). * Indicates significant difference in VK content in mature milk from 0–2 months and 3–5 months after delivery.

**Table 5:** Recommended AI values for infants aged 0–5 months.

Month	Average Mature Milk Intake (mL/d)	Country	TVK (ng/mL)	VK1 (ng/mL)	VK2 (ng/mL)	AI of VK1 (μg/d)	AI of VK2 (μg/d)	AI of VK (μg/d)
0–5	776.80 [7]	China, 2023	4.50 (2.84–6.35)	2.81 (1.65–4.41)	1.37 (0.75–2.11)	2.18	1.06	3.49
0–6	679.60–825.20 [8]	WHO, 2002	4.50 (2.85–6.33)	2.81 (1.66–4.39)	1.37 (0.75–2.11)	1.91–2.32	0.93–1.13	3.05–3.71
1–6	765.00 [9]	Australia, 2006	4.50 (2.84–6.35)	2.81 (1.65–4.41)	1.37 (0.75–2.11)	2.15	1.05	3.44
2–6	862.10 [10]	Bolivia, 2018	4.57 (2.65–6.73)	3.08 (1.71–4.92)	1.14 (0.60–1.90)	2.65	0.98	3.94
2	746.00 [11]	Indonesia, 2020	4.80 (2.99–6.97)	3.06 (1.98–4.99)	1.14 (0.64–2.23)	2.29	0.85	3.58
3	884.00 [12]	Bangladesh, 2007	4.68 (2.88–6.83)	3.32 (1.82–5.48)	1.28 (0.56–1.92)	2.93	1.13	4.14
	753.70 [13]	Australia, 2021				2.50	0.96	3.53
	796.10 [14]	Brazil, UK, etc., 2010				2.64	1.02	3.73
5	701.00 [11]	Indonesia, 2020	4.49 (2.86–7.21)	2.94 (2.10–5.41)	1.26 (0.53–1.97)	2.06	0.88	3.14
6	874.80 [15]	Iceland, 2012	3.98 (3.47–)	3.14 (2.33–)	0.85 (0.54–)	2.75	0.74	3.48

AI (μg/d) = mature milk intake (mL/d) × VK concentration (ng/mL) × 10^−3^. Mature milk intake has been transformed into a common unit (mL/day) using the conversion 1.03 g/mL [16]. VK concentration is expressed as P50 (P25–P75).

## Data Availability

The data not published within this article are available from the corresponding author upon reasonable request.
